# Korrelation zwischen Stimulationsleveln und ECAP-Schwellen bei audiometriebasierter Anpassung in der Cochleaimplantatversorgung

**DOI:** 10.1007/s00106-025-01584-6

**Published:** 2025-04-07

**Authors:** Annett Franke-Trieger, Thomas Hocke, Thomas Zahnert, Susen Lailach

**Affiliations:** 1 Sächsisches Cochlear Implant Center, Klinik und Poliklinik für Hals- Nasen- und Ohrenheilkunde, Universitätsklinikum Dresden, Medizinische Fakultät, Technische Universität Dresden, Fetscherstraße 74, 01307 Dresden, Deutschland; 2 Cochlear Deutschland GmbH & Co. KG, Hannover, Deutschland

**Keywords:** Cochleaimplantat, Audiometrie, Elektrisch evozierte Summenaktionspotenziale, Prädiktion, Sprachaudiometrie, Cochlear implant, Audiometry, Electrically evoked compound action potentials, Prediction, Speech audiometry

## Abstract

**Hintergrund:**

Patienten mit noch messbarem maximalem Einsilberverstehen, mEV, und unzureichendem Sprachverstehen mit Hörgerät bei 65 dB_SPL_, EV_65_(HG), können von einem Cochleaimplantat (CI) profitieren. Anhand eines validierten Prädiktionsmodells lässt sich für diese Patientengruppe das Sprachverstehen mit CI, EV_65_(CI), abschätzen. Ziel der Studie ist die Untersuchung der Korrelation zwischen elektrisch evozierten Summenaktionspotenzialen und Stimulationsparametern des CI-Systems.

**Material und Methoden:**

In der prospektiven Studie wurden 37 CI-versorgte Patienten eingeschlossen. Das EV_65_(CI) wurde nach 6 und 12 Monaten bestimmt und mit dem prognostiziertem EV_65_(CI) verglichen. Die Korrelation von minimaler und maximaler elektrischer Stimulation mit den Summenaktionspotenzialen wurde untersucht.

**Ergebnisse:**

Bei allen Patienten zeigt sich ein signifikant besseres EV_65_(CI) nach 12 Monaten im Vergleich zum präoperativ ermittelten EV_65_(HG). Der Anteil der Patienten, welche die Prädiktion um mehr als 20 Prozentpunkte (pp) verfehlten, lag nach 6 Monaten bei 19 % und nach 12 Monaten bei 5 %. In der Patientenpopulation zeigte sich eine höhere Korrelation der Schwellen der Summenaktionspotenziale mit der maximalen als mit der minimalen elektrischen Stimulation.

**Schlussfolgerung:**

Die CI-Versorgung von Patienten mit unzureichendem Sprachverstehen mit Hörgerät – auch mit einem sehr hohen präoperativen maximalen Einsilberverstehen bei mäßig schwerem Hörverlust – stellt eine erfolgversprechende Therapieoption dar. Das Prädiktionsmodell ermöglicht eine individuelle Planung der Therapie. Die Anwendung des Modells trug zu einer höheren Versorgungsqualität im Vergleich zu einer früheren retrospektiven Studie bei. Die gefundene Korrelation zwischen Stimulationsintensität und Summenaktionspotenzialschwellen in einer Patientenpopulation mit erreichter Prädiktion unterstützt die These, dass eine ECAP- („evoked compound action potentials“) und audiometriebasierte Anpassung ein erfolgreicher Ansatz ist.

## Abstract (en)

**Background:**

Persons whose speech recognition with hearing aids at 65 dB_SPL_, WRS_65_(HA), is inadequate but whose maximum monosyllabic word recognition score (WRS_max_) is still measurable may benefit from a cochlear implant (CI). Using a validated prediction model, one can estimate word recognition with a CI, WRS_65_(CI), for these patients. The aim of this study was to investigate the relationship between electrically evoked compound action potentials (ECAP) and stimulation parameters of the CI system.

**Patients and methods:**

In this prospective study, 37 patients were included. The WRS_65_(CI) was determined after 6 and 12 months and compared with the predicted WRS_65_(CI). The correlation of minimum and maximum electrical stimulation with the ECAP was investigated.

**Results:**

All patients showed a significantly better WRS_65_(CI) after 12 months compared with the preoperatively determined WRS_65_(HA). The proportion of patients whose WRS_65_(CI) fell short of the predicted value by more than 20 percentage points was 19% after 6 months and 5% after 12 months. In the patient population, there was a closer correlation of the ECAP thresholds with the maximum than with the minimum electrical stimulation.

**Conclusion:**

For patients whose speech understanding with hearing aids is inadequate—even with a very high preoperative WRS_max_ in moderately severe hearing loss—CI fitting appears to be a promising treatment option. The predictive model enables individual treatment planning. Its application contributed to a quality of care that was higher than was found in an earlier retrospective study. The correlation found between stimulation intensity and ECAP in a cohort of patients with achieved predicted values supports the hypothesis that a fitting procedure based on ECAP and audiometry is a promising approach.

Wenn schwerhörige Patienten mit einem Hörgerät kein ausreichendes Sprachverstehen erreichen, sollte die Versorgung mit einem Cochleaimplantat (CI) in Betracht gezogen werden [9]. In den letzten Dekaden zeigte sich eine medizinisch-audiologische Erweiterung in der Indikationsstellung zur CI-Versorgung. Während anfänglich nur Patienten mit kompletter beidohriger Taubheit versorgt worden sind [6, 27], werden aktuell auch Patienten mit präoperativ hohem maximalem Einsilberverstehen, mEV, mit einem CI versorgt [7, 12, 19, 33, 36, 39], wenn trotz optimierter Hörgeräteversorgung kein ausreichendes Sprachverstehen zu erreichen ist. Ein Referenzpunkt für die audiologische Indikationsstellung ist das seitengetrennt gemessene Einsilberverstehen in Ruhe bei 65 dB_SPL_ mit optimierter Hörgeräteeinstellung, EV_65_(HG). Die audiologische CI-Indikation ist erfüllt, wenn das EV_65_(HG) ≤ 60 % beträgt [9]. Diese Messkondition wird konsequenterweise auch für die regelmäßige postoperative Kontrolle des CI-Systems empfohlen [8] und wird dementsprechend auch als EV_65_(CI) im nationalen CI-Register [35] abgebildet.

Aufgrund der hohen Variabilität des EV_65_(CI) war in den letzten Jahrzehnten eine evidenzbasierte Definition individueller audiometrischer Rehabilitationsziele nicht möglich. Vielmehr basierte die Zieldefinition auf Erfahrung der Zentren und dem Wissen um publizierte Sprachtestergebnisse inhomogener Patientengruppen [4, 14]. Entsprechend schwierig war es, Patienten mit nicht zufriedenstellendem Rehabilitationsverlauf zu identifizieren, um möglichst frühzeitig therapeutische und audiologische Maßnahmen einzuleiten. Insbesondere bei resthörigen Patienten haben die Parameter mEV und EV_65_(HG) an Bedeutung gewonnen [21], da in dieser Patientengruppe bei nicht zufriedenstellendem Verlauf auch eine Verschlechterung der Hörsituation nach der Implantation denkbar ist [7, 12, 19, 20, 33, 36, 39].

Ein Modell, Gl. 1, welches die Relationen zwischen prä- und postoperativem Einsilberverstehen, mEV, EV_65_(HG) und EV_65_(CI), sowie dem Lebensalter zum Zeitpunkt der CI-Versorgung [21] nutzt, ermöglicht eine individuelle Abschätzung der möglichen Verbesserung.

1 $$\begin{aligned}& EV_{65}\left(CI\right)\left[{\%}\right]\\&=\frac{100}{1+e^{-\left(\beta _{0}+\beta _{1}\cdot mEV+\beta _{2}\cdot age+\beta _{3}\cdot EV_{65}\left(HG\right)\right)}}\end{aligned}$$

mit β_0_ = 0,84 ± 0,18; β_1_ = 0,012 ± 0,0015 [1/%]; β_2_ = −0,0094 ± 0,0025 [1/Jahr]; β_3_ = 0,0059 ± 0,0026 [1/%].

Hoppe et al. [18] haben in einer Evaluierungsstudie sogar bessere Werte für das EV_65_(CI) gefunden und argumentieren, dass diese Verbesserung durch den fortlaufenden Abgleich zwischen erreichtem und prognostiziertem EV_65_(CI) während der postoperativen Rehabilitation erreicht wurde. Bei Patienten, für die das erreichte vom prognostizierten EV_65_(CI) zunächst abwich, wurde eine frühe Intervention im Rehabilitationsprozess durchgeführt, was eine Verbesserung des Sprachverstehens zur Folge hatte. In weiteren Studien mit anderer originärer Fragestellung wurde das Modell alio loco retrospektiv bestätigt [7, 12, 39]. Aktuelle Studien von vier deutschen CI-versorgenden Einrichtungen an insgesamt 850 Patienten zeigen einen Prädiktionsfehler („median absolute error“, MAE) für das EV_65_(CI) zwischen 9,9 und 13,5 Prozentpunkten (pp) [7, 12, 18, 19, 21, 39].

In einer Studie von Dziemba et al. [11] wurde die Abweichung zur Prognose für die systematische Analyse schlechten Sprachverstehens mit dem CI genutzt: Patienten, die weit hinter der Prognose zurückblieben, zeigten signifikant schlechtere Werte in der kategorialen Lautheitsskalierung (KLS) und dem Hörverlust für Zahlen (HVZ). Außerdem zeigten sich Auffälligkeiten in der Sprachdiskriminationsfunktion und beim Abgleich der eingestellten elektrischen Hörschwellen, mit der die minimale Stimulationsintensität festgelegt wird (T-Level) mit den von Rader et al. empfohlenen Werten [32]. Ein großer Teil (55 %) der Variabilität im EV_65_(CI) kann somit durch prinzipiell beeinflussbare Einstellungen des CI-Systems erklärt werden. Diese Verfahren sowie der Abgleich mit objektiven Methoden [2, 5, 10, 13, 22, 23] sind in unserer Einrichtung Bestandteil der klinischen Routine. So stellt die Messung der über das CI auslösbaren Summenaktionspotenziale, „electrically evoked compound action potential“, ECAP, einen weiteren wichtigen Bestandteil der CI-Diagnostik dar [22, 23, 25]. Neben der Lagebeziehung zwischen CI-Elektrode und Modiolus [30] hat auch der präoperative Hörstatus Einfluss auf die gemessenen ECAP-Schwellen, tECAP [28]. Auch kommen nicht alle Studien zu einem einheitlichen Ergebnis hinsichtlich des Nutzens der tECAP für die Einstellung der CI-Systeme [15, 25, 29, 31].

Ziel dieser Studie ist die Untersuchung der Beziehungen von den tECAP zu den minimalen und maximalen Stimulationsintensitäten, T‑ und C‑Leveln, bei Patienten mit einem mEV > 0. Insbesondere wird untersucht, inwieweit die Berücksichtigung des Zielkorridors für das EV_65_(CI) und die daraufhin ausgerichtete Optimierung der tECAP- und audiometriebasierten Anpassung der T‑ und C‑Level (Fitting) diese Lagebeziehungen beeinflusst.

## Patienten und Methoden

Die Datenerhebung erfolgte im Rahmen der CI-Vordiagnostik sowie der sich anschließenden Basis- und Folgetherapie. Sämtliche Untersuchungen erfolgten am Sächsischen Cochlear Implant Centrum der Klinik und Poliklinik für Hals-Nasen-Ohrenheilkunde am Universitätsklinikum Dresden. Die prospektive Studie wurde von der Ethikkommission an der TU Dresden befürwortet (SR+BO-260052021). Eine Registrierung erfolgte beim Deutschen Register für Klinische Studien (DRKS00026741). Die Daten der Studie können bei Bedarf zur Verfügung gestellt werden.

### Einschlusskriterium und Patientenkollektiv

Es wurden die Daten von Patienten ausgewertet, welche zwischen dem 01.07.2021 und 05.04.2023 mit dem Implantattyp Cochlear^TM^ Nucleus® Profile Plus mit Slim Straight Elektrodenträger (CI622) und den Sprachprozessoren CP1000/CP1110/CP1150 (Fa. Cochlear Ltd., Sydney, Australien) versorgt worden sind. Die Indikationsstellung erfolgte entsprechend der aktuellen deutschen AWMF-Leitlinie [9]. Im Studienzeitraum erfolgten 122 Cochleaimplantationen mit dem CI622. Nach Ausschluss der pädiatrischen Fälle verblieben 92 Patienten, von denen weitere 56 Patienten aus den folgenden Gründen ausgeschlossen wurden:

- mEV = 0 (36)
- prälinguales Einsetzen einer hochgradigen Schwerhörigkeit (5)
- Muttersprache nicht Deutsch (3)
- Ertaubungsursache Meningitis (2)
- kognitive Einschränkung (3)
- Z. n. Vestibularisschwannom-Operation (1)
- Z. n. Felsenbeinfraktur (1)
- unvollständige Insertion (1)
- Umgeknickte Elektrodenspitze, „tip fold-over“ (1)
- vorzeitiger Abbruch der Rehabilitationsmaßnahmen (2)

Somit konnten 37 Patienten in die Studie eingeschlossen werden. Bei 33 Patienten handelte es sich um eine ipsilaterale Erstversorgung, bei 4 Patienten um eine Versorgung des zweiten Ohrs. Das Kollektiv bestand aus 21 Frauen und 16 Männern. Das Alter der Patienten zum Zeitpunkt der Implantation lag zwischen 18 Jahren und 84 Jahren bei einem Mittelwert von 63 ± 16 Jahren.

### Audiologische Zielparameter

Folgende Daten wurden ausgewertet: präoperativ unilateral durchgeführter Freiburger Einsilbertest mit und ohne Hörgerät: EV_65_(HG), mEV; präoperatives Reintonaudiogramm, Alter bei der Implantation, Freiburger Einsilbertest mit CI – sechs und zwölf Monate nach Implantation: EV_65_(CI); T und C-Level, tECAP zwölf Monate nach Implantation. Aus dem präoperativen Reintonaudiogramm wurde der Hörverlust als Mittelwert der Hörschwelle für die Frequenzen 0,5; 1; 2 und 4 kHz berechnet (4FPTA, „four-frequency pure tone average“). Bei Erreichen der Messgrenze von 120 dB_HL_ wurde der 4FPTA mit einem Wert von 120 dB_HL_ berechnet.

### Datenerhebung und Statistik

Alle Messungen erfolgten in einer Audiometriekabine (DIN EN ISO 8253). Die präoperative unversorgte Testung (4FPTA, mEV) erfolgte mit Kopfhörern. Zur Ermittlung des mEV wurde der Darbietungspegel in 10-dB-Schritten erhöht, bis ein Darbietungspegel nahe, aber unterhalb der Unbehaglichkeitsschwelle erreicht war. Die sprachaudiometrischen Messungen mit Versorgung, EV_65_(HG) und EV_65_(CI), erfolgten im Freifeld, wobei der Patient 1 m vor dem Lautsprecher platziert wurde. Wenn notwendig, wurde das Gegenohr regelrecht geblockt bzw. vertäubt. Genutzt wurde ein Audiometer des Typs AT900 oder AT1000 (Fa. Auritec GmbH, Hamburg, Deutschland). Die Prognose für das EV_65_(CI) nach 6 Monaten wurde nach Gl. 1 berechnet. Der Vorhersagefehler wurde über den medianen absoluten Fehler (MAE) sowie den medianen Fehler (ME) quantifiziert. Der MAE wird verwendet, um die Genauigkeit von Prognosen zu bestimmen. Da in die Berechnung des MAE ausschließlich die absolute Abweichung der Prognose vom tatsächlich gemessenen Wert eingeht, beschreibt er die Höhe, nicht jedoch die Richtung der Abweichung. Der ME ist ein Maß für die Richtung der Abweichung, da hierfür ein Mittelwert aus den Abweichungen inkl. Vorzeichen berechnet wird. Signifikante Differenzen zwischen den sprachaudiometrischen Testergebnissen wurden nach Winkler und Holube [40] ermittelt. Die Korrelationsanalysen erfolgten über die Spearman-Ranganalyse. Der Vergleich der Korrelationskoeffizienten erfolgte über die z‑Transformation nach Fisher: Überlappen sich die 95%-Konfidenzintervalle zweier Korrelationskoeffizienten nicht, sind diese signifikant voneinander verschieden. Die Datenanalyse und Erstellung der Abbildung erfolgte mit den Softwarepaketen OriginLab (Version 2019, Fa. OriginLab, Northampton/MA, USA) und MATLAB® (Version 2019b, Fa. MathWorks, Natick/MA, USA).

### Anpassung des CI-Systems und ECAP-Messungen

In der CI-versorgenden Einrichtung der Autoren erfolgt die Einstellung von CI-Systemen der Fa. Cochlear i. d. R. ECAP-basiert [3, 5, 22, 23]. ECAP-basiert bedeutet, dass sich bei der Einstellung der C‑Level weitgehend an den tECAP orientiert wird. Hierbei erfolgt zunächst eine Einzelkanalstimulation. Die daraus resultierende Einstellung wird anschließend im Live-Modus getestet. Die tECAP sind also in diesem Prozess orientierend für eine erste Einstellung der Absolutwerte der C‑Level sowie deren Profil. Die ECAP werden im Verlauf regelmäßig (mindestens einmal im Jahr) gemessen.

Eine deutlich abweichende Einstellung der C‑Level von den tECAP wird bei folgenden Symptomen mit Verdacht auf Über- oder Unterstimulation vorgenommen:

- Diskriminationsfunktion für Einsilber: der Wert für das EV(CI) bei 80 dB liegt erheblich über oder unter dem EV_65_(CI). Als Vergleich dienen Ergebnisse an langzeitversorgten CI-Trägern für Pegel von 60–80 dB_SPL_ [16]
- Subjektive Angaben, welche durch zusätzliche Messungen (z. B. KLS) bestätigt werden
- Stapediusreflexschwellen weichen deutlich von Erwartungswerten ab [13, 31]

Besonderer Wert wird auf die Anpassung der T‑Level gelegt. Diese folgen i. d. R. zunächst in ihrem Profil den tECAP bzw. C‑Leveln bei einer voreingestellten Dynamik von ca. 50 Current Level (cL). Current Level ist ein herstellerspezifisches Maß für die Stimulationsintensität. Sie ist über die Gl. 2 mit der Stromstärke verknüpft.

2 $$\mathrm{I}\left[\mathrm{\mu}A\right]=17{,}5*100^{\frac{\mathrm{cL}}{255}}$$

Im weiteren Verlauf werden die T‑Level durch Einzelkanalstimulation überprüft und ggf. verändert [32]. Ein weitere funktionelle Kontrolle erfolgt über die Bestimmung des HVZ [11].

Die Registrierung der ECAP erfolgte mit der Auto-NRT-Funktion der Software Custom Sound Pro mit einer Pulsbreite von 25 µs. Waren derart keine Potenziale ableitbar, wurde mit der Software Custom Sound EP bei höheren Pulsweiten (37–50 µs) gemessen. Zur Bestimmung der tECAP wurden die gemessenen Potenziale mit der Software Custom Sound EP ausgewertet. Dabei erfolgte die Entscheidung über das Vorhandenseins eines Potenzials entsprechend den Arbeiten von Hey et al. und Müller et al. [17, 30]: Amplituden kleiner als 10 µV wurden nicht als ECAP bewertet. Amplituden größer oder gleich 10 µV wurden als ECAP bewertet.

## Ergebnisse

### Präoperative Audiometrie

**Abb. 1:**
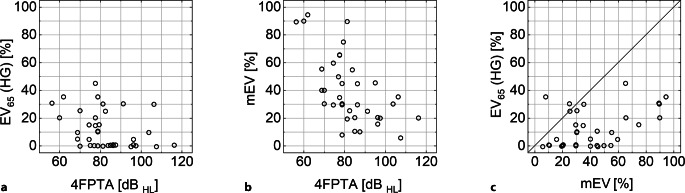
Ergebnisse der präoperativen Ton- und Sprachaudiometrie für die 37 Studienteilnehmer. **a** Einsilberverstehen mit Hörgerät, EV_65_(HG), über dem mittleren Hörverlust, 4FPTA; **b** maximales Einsilberverstehen, mEV, über dem 4FPTA. **c** Zusammenhang zwischen mEV und dem EV_65_(HG)

In Abb. 1 sind die Ergebnisse der präoperativen audiometrischen Untersuchungen dargestellt. Das EV_65_(HG) lag zwischen 0 und 45 % bei einem 4FPTA zwischen 56 und 116 dB_HL_. Entsprechend dem Einschlusskriterium für diese prospektive Studie lag das mEV bei allen Patienten oberhalb 0 % und erreichte Werte von bis zu 95 %. In der Mehrzahl der Patienten lag das EV_65_(HG) unterhalb des mEV, siehe Winkelhalbierende in Abb. 1c.

### Postoperative vs. präoperative Audiometrie

**Abb. 2:**
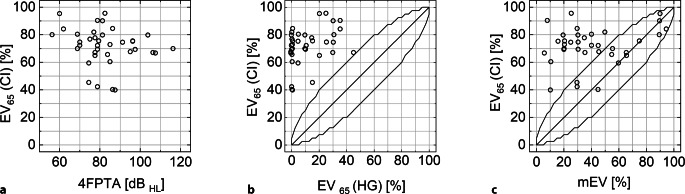
Ergebnisse der prä- und postoperativen Audiometrie der 37 Studienteilnehmer nach 12 Monaten. **a** Einsilberverstehen mit CI, EV_65_(CI), über dem mittleren präoperativen Hörverlust, 4FPTA. **b** EV_65_(CI) über dem präoperativen Einsilberverstehen mit Hörgerät, EV_65_(HG). **c** EV_65_(CI) über dem präoperativen maximalen Einsilberverstehen, mEV

Die Abb. 2 zeigt das EV_65_(CI) nach 12 Monaten in Relation zum präoperativen 4FPTA, EV_65_(HG) und mEV. Für alle Patienten konnte eine signifikante Verbesserung des EV_65_(CI) gegenüber dem präoperativen EV_65_(HG) beobachtet werden. Das EV_65_ verbesserte sich von 5 % mit dem Hörgerät auf 72,5 % mit dem CI. Bei 23 Patienten (62 %) lag das EV_65_(CI) signifikant über dem mEV. Bei keinem Patienten wurde das mEV signifikant unterschritten.

### Postoperatives Sprachverstehen und Abweichung von der Prognose

**Abb. 3:**
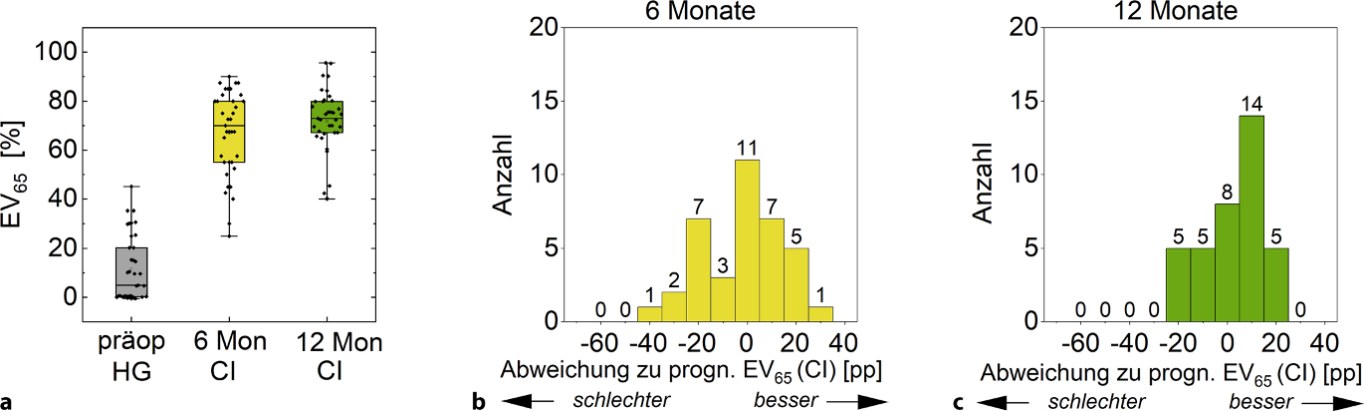
**a** Zeitlicher Verlauf des Einsilberverstehens mit CI, EV_65_(CI), nach sechs und zwölf Monaten im Vergleich zu den mit Hörgerät erzielten Werten, EV_65_(HG), sowie (**b** und **c**) die Abweichung vom prognostizierten EV_65_(CI) zu den ausgewerteten Zeitpunkten

In Abb. 3 ist der zeitliche Verlauf des EV_65_ sowie der Vergleich zur Prognose nach Gl. 1 dargestellt. Daraus ergibt sich ein MAE nach sechs Monaten von 10,5 pp und nach zwölf Monaten von 9,6 pp. Der ME liegt nach sechs Monaten bei −0,7 pp, d. h., im Median wurde die Prognose um 0,7 pp verfehlt. Nach zwölf Monaten liegt der ME bei 7,4 pp, d. h., die Prognose wurde um 7,4 pp übertroffen.

### Analyse der Stimulationsparameter

**Abb. 4:**
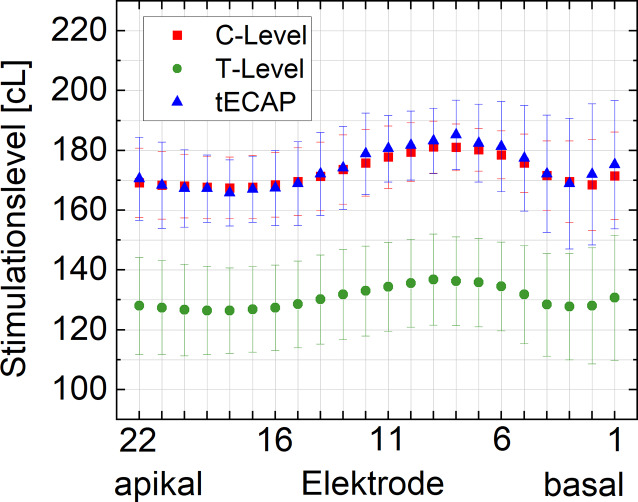
Minimale und maximale Stimulationspegel der Prozessoreinstellungen, T‑ bzw. C‑Level, und deren Beziehung zu den Schwellen für die Summenaktionspotenziale, tECAP

**Abb. 5:**
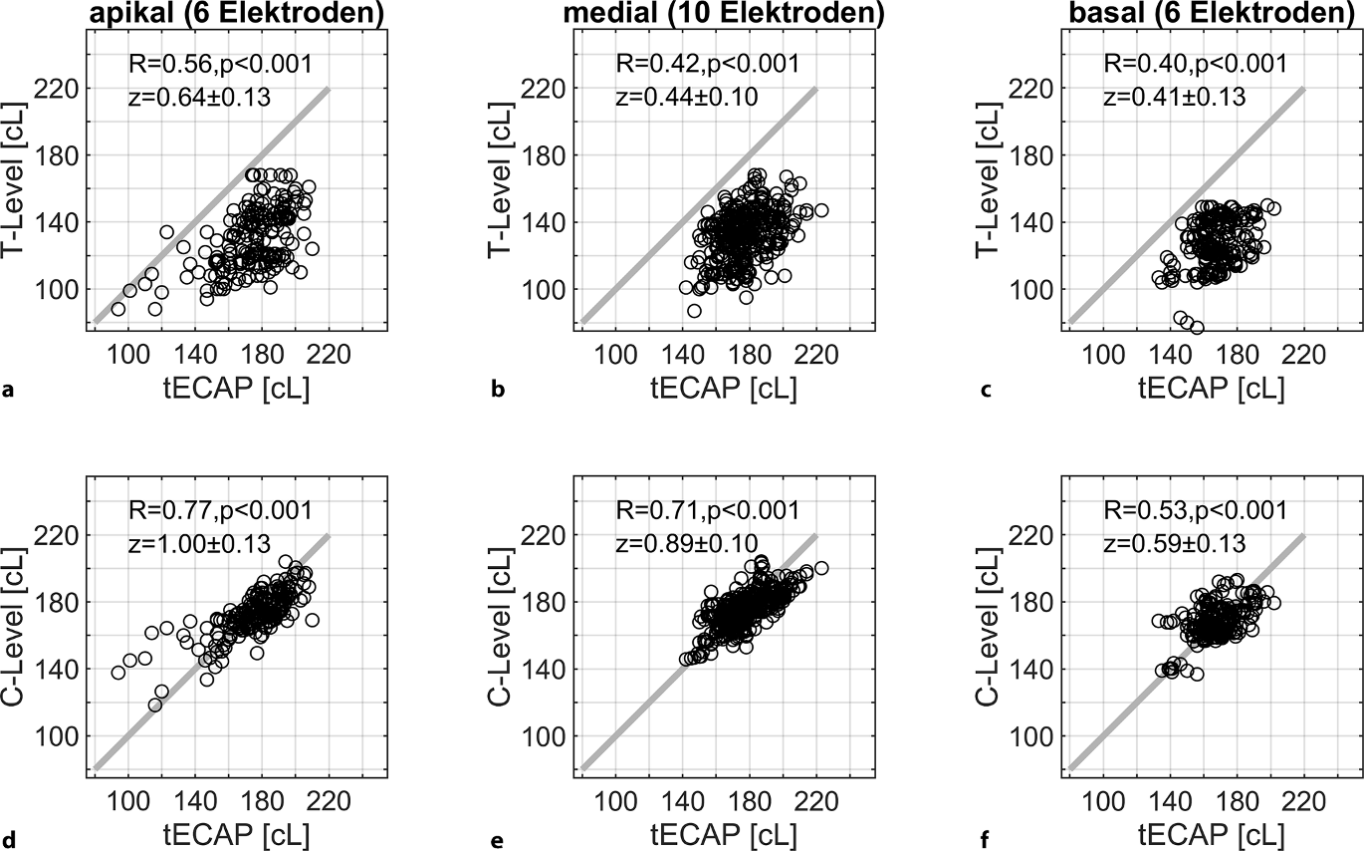
Korrelation der minimalen und maximalen Stimulationspegel, **a–c** T- und **d–f** C-Level, der Prozessoreinstellungen zu den Schwellen der Summenaktionspotenziale, tECAP. Die apikalen (E22–E17), medialen (E16–E7) und basalen Bereiche (E6–E1) wurden hier getrennt betrachtet. Neben den Korrelationskoeffizienten R sind die z‑Transformierten nach Fisher sowie deren 95%-Konfidenzintervalle dargestellt

In Abb. 4 sind die Mittelwerte und die einfachen Standardabweichungen der T‑ bzw. C‑Level sowie der gemessenen tECAP nach 12 Monaten dargestellt. Die mittleren C‑Level liegen zwischen 167 ± 11 cL für Elektrode 17 und 181 ± 9 cL für Elektrode 9. Der mittlere elektrische Dynamikbereich liegt zwischen 40 cL und 45 cL. Die mittleren C‑Level folgen in ihrem elektrodenabhängigen Verlauf den tECAP. Die entsprechende Korrelationsanalyse erfolgte für den apikalen (E22–E17), medialen (E16–E7) und basalen Bereich (E6–E1) getrennt. In Abb. 5 sind jeweils die T‑Level (Abb. 5a–c) und die C‑Level (Abb. 5d–f) in ihrer Relation zu den tECAP aufgetragen. Für die C‑Level finden sich für den apikalen und medialen Bereich höhere Korrelationen zu den tECAP als für die T‑Level. Auch ist die Korrelation der C‑Level mit den tECAP apikal und medial höher als basal.

## Diskussion

Es wurde anhand eigener Daten ein Modell [21] zur Prädiktion des EV_65_(CI) evaluiert. Zusätzlich zu den bisher erfolgten Evaluationsstudien [7, 11, 12, 18, 39] erfolgte eine Analyse der Stimulationspegel und ECAP-Schwellen. Die Prädiktion hatte im Studienzeitraum direkten Einfluss auf die Folgetherapie im Versorgungsprozess. Bei Patienten mit verfehlter Prädiktion wurde frühzeitig interveniert. Derart konnte nach zwölf Monaten bei allen Patienten eine signifikante Verbesserung des EV_65_(CI) gegenüber dem EV_65_(HG) beobachtet werden. Die Prädiktion wurde im Median nach 12 Monaten übertroffen, wohingegen sie nach 6 Monaten noch knapp verfehlt wurde.

### Modellevaluation, Vorhersagefehler sowie Qualitätssicherung in der Basis- und Folgetherapie

Der MAE für das EV_65_(CI) liegt für 6 Monate mit 10,5 pp im Bereich anderer Studien mit Werten von 9,9 bis 13,5 pp [7, 13, 18, 19, 21, 39]. Allerdings sollte neben dem MAE auch der ME betrachtet werden, da sich der MAE auch bei zu hohen (guten) EV_65_(CI) erhöht. So liegt der MAE für die Jahreswerte zwar nur noch bei 9,6 pp, jedoch tragen hierzu größtenteils die die Prognose übertreffenden Werte bei; wie es der ME von 7,4 pp herausstellt. Das genutzte Prädiktionsmodell ist also geeignet, um Patienten mit *unerwartet schlechtem Sprachverstehen* (Prognose um mindestens 20 pp verfehlt, [18]) frühzeitig zu identifizieren und dem folgend multidisziplinäre Maßnahmen in der CI-Folgetherapie einzuleiten. Hierzu zählen u. a. neben der Abklärung möglicher pathophysiologischer Ursachen eine Kontrolle der Integrität des CI-Systems und eine systematische Analyse der Stimulationsparameter [11, 18]. Diese Vorgehensweise hat in der hier vorliegenden und einer anderen prospektiven Studie [18] zu einer Verbesserung der Ergebnisse im jeweiligen Vergleich zu den beiden retrospektiven Analysen beigetragen; in domo [12] und Hoppe et al. [21]. Jedoch verfehlten 7 von 37 Patienten (19 %) nach sechs Monaten ihre Prädiktion um mindestens 20 pp. Nach 12 Monaten traf dies nur noch auf 2 von 37 Patienten (5 %) zu. Dies entspricht der von Hoppe et al. [18] angegebenen Größenordnung von 16 % nach 6 Monaten bzw. 6 % nach 12 Monaten. Bemerkenswert ist hierbei, dass die 7 Patienten mit *unerwartet schlechtem Sprachverstehen* nach 6 Monaten eine Verbesserung gegenüber dem EV_65_(HG) von 10, 15, 25, 30, 40, 40 und 45 pp erreichten: d. h., bei diesen Patienten wären ohne eine individuelle Erwartungshaltung seitens der versorgenden Einrichtung die Schwerpunkte möglicherweise anderweitig gelegt worden.

### Anpassung des CI-Systems

Bei der Anpassung des CI-Systems handelt es sich um einen zeitaufwendigen Prozess, bei dem die angewendeten Methoden zwischen einzelnen Zentren und Mitarbeitern differieren können [37]. Während in den Anfängen der CI-Versorgung das Fitting nur von subjektiven Angaben abhing, wurden in den letzten Jahren zunehmend Qualitätsindikatoren für die Bewertung des Fittings erarbeitet [11, 13]. Derzeit werden z. B. objektive Methoden wie die Stapediusreflexmessung genutzt [13]. Außerdem wurde die Wertigkeit der ECAP für die Kontrolle des Fittings diskutiert, ohne jedoch eine abschließende Bewertung vornehmen zu können [38]. Insgesamt finden sich herstellerübergreifend teilweise widersprüchliche Ergebnisse [1, 24, 26, 41]. Während Lambriks et al. beispielsweise ebenfalls eine starke Korrelation der C‑Level mit den tECAP für das Implantat HiRes Ultra der Fa. Advanced Bionics zeigten [26], konnten de Vos et al. im Rahmen einer herstellerübergreifenden Metaanalyse nur eine moderate Korrelation der C‑Level mit den tECAP ermitteln [38].

Im Unterschied zu einer älteren Arbeit [25] finden sich in unserer Arbeit für den Zusammenhang zwischen den T‑Leveln und den tECAP niedrigere Korrelationen als für den Zusammenhang zwischen den C‑Leveln und den tECAP. Die ersten Einstellungen des CI-Systems erfolgten bei uns ECAP-basiert, d. h., die C‑Level orientieren sich absolut an den tECAP, während die T‑Level dem Profil folgen. Daraus folgt zwangsläufig eine Korrelation von T‑ und C‑Leveln mit den tECAP von R = 1. Beide Stimulationspegel werden, wie im Methodenteil beschrieben, basierend auf der audiologischen Diagnostik (Sprachaudiometrie, Schwellenbestimmung, Lauheitsskalierung) kontrolliert und nachjustiert. Jedoch sind die T‑Level stärker von notwendigen Änderungen betroffen als die C‑Level; ein in dieser Arbeit erstmalig beschriebener Umstand. Bei erfolgreicher CI-Basistherapie (Prognose wird erreicht) und dementsprechender Nachjustierung der Prozessoreinstellungen – insbesondere im schwellennahen Bereich – verringert sich die Korrelation der T‑Level mit den tECAP. Zusätzlich ist die hohe Plastizität der Spiralganglienneurone zu beachten: Schwitzer et al. konnten zeigen, dass sich bei zu hoch eingestellten C‑Leveln die tECAP zu höheren Pegeln hin verschieben [34]. Daraus ergäbe sich wiederum eine höhere Korrelation beider Größen.

Aufgrund der in dieser Studie dargestellten Korrelation ist die Einbeziehung der tECAP und deren Abgleich mit den C‑Leveln zur Bewertung der Einstellung, zumindest des in dieser Studie analysierten CI-Systems, empfehlenswert. Vor allem bei Patienten mit unterschrittener Prognose sollte nicht nur ein alleinstehender Abgleich der tECAP mit der subjektiv ermittelten Stimulationsintensität, sondern auch ein Abgleich mit den Ergebnissen der Funktionsdiagnostik erfolgen, insbesondere um die T‑Level zu optimieren. Die hier dargestellten Zusammenhänge lösen u. E. einen Teil der kontrovers geführten Diskussion [15, 25, 29, 31] über das Für und Wider der ECAP-basierten Anpassung auf: Die in Abb. 4 dargestellten Mittelwerte suggerieren im Mittel eine sehr hohe Korrelation der T‑ und C‑Level mit den tECAP. Erst die nachfolgende, detaillierte Analyse der Stimulationsparameter (Abb. 5) und der erfolgreiche Prozess der Anpassung mit erreichter Prognose verdeutlichen, dass die ECAP-basierte Anpassung ein effizienter Weg für eine erste Einstellung des CI-Systems ist, jedoch bei den meisten Patienten auch eine individuelle Nachjustierung erfordert.

## Fazit für die Praxis

- Die leitlinienkonforme CI-Versorgung von Patienten mit unzureichend versorgtem Sprachverstehen – auch mit einem sehr hohen präoperativen maximalen Einsilberverstehen bei mäßig schweren Hörverlust – stellt eine erfolgversprechende Therapieoption dar.
- Das genutzte Prädiktionsmodell ermöglicht eine bedarfsgerechte und individuelle Planung der Therapieeinheiten sowie der audiologischen Maßnahmen während der CI-(Re‑)Habilitation.
- Der mediane absolute Fehler, der mediane Fehler und der Anteil der Patienten, die die Prognose um 20 pp oder mehr verfehlen, sind neben anderen Endpunkten geeignete Parameter zum Qualitätsmonitoring innerhalb der Basis- und Folgetherapie.
- In dieser prospektiven Studie beobachteten wir eine Steigerung der Versorgungsqualität im Vergleich zu einer früheren retrospektiven Studie.
- Folglich trug die Anwendung des Modells in Verbindung mit der ECAP- und audiometriebasierten Anpassung des CI-Systems zu einer verringerten Anzahl von Fällen mit *unerwartet schlechtem Sprachverstehen* bei.

## Data Availability

Die Daten, auf denen die Ergebnisse dieser Studie basieren, sind auf Anfrage beim korrespondierenden Autor erhältlich.
